# Retrieval of Context-Associated Memory is Dependent on the Ca_v_3.2 T-Type Calcium Channel

**DOI:** 10.1371/journal.pone.0029384

**Published:** 2012-01-03

**Authors:** Chien-Chang Chen, Jhe-Wei Shen, Ni-Chun Chung, Ming-Yuan Min, Sin-Jong Cheng, Ingrid Y. Liu

**Affiliations:** 1 Institute of Biomedical Sciences, Academia Sinica, Taipei, Taiwan; 2 Department of Molecular Biology and Human Genetics, Tzu Chi University, Hualien, Taiwan; 3 Institute of Zoology, National Taiwan University, Taipei, Taiwan; Case Western Reserve University, United States of America

## Abstract

Among all voltage-gated calcium channels, the T-type Ca^2+^ channels encoded by the Ca_v_3.2 genes are highly expressed in the hippocampus, which is associated with contextual, temporal and spatial learning and memory. However, the specific involvement of the Ca_v_3.2 T-type Ca^2+^ channel in these hippocampus-dependent types of learning and memory remains unclear. To investigate the functional role of this channel in learning and memory, we subjected Ca_v_3.2 homozygous and heterozygous knockout mice and their wild-type littermates to hippocampus-dependent behavioral tasks, including trace fear conditioning, the Morris water-maze and passive avoidance. The Ca_v_3.2 ^−/−^ mice performed normally in the Morris water-maze and auditory trace fear conditioning tasks but were impaired in the context-cued trace fear conditioning, step-down and step-through passive avoidance tasks. Furthermore, long-term potentiation (LTP) could be induced for 180 minutes in hippocampal slices of WTs and Ca_v_3.2 ^+/−^ mice, whereas LTP persisted for only 120 minutes in Ca_v_3.2 ^−/−^ mice. To determine whether the hippocampal formation is responsible for the impaired behavioral phenotypes, we next performed experiments to knock down local function of the Ca_v_3.2 T-type Ca^2+^ channel in the hippocampus. Wild-type mice infused with mibefradil, a T-type channel blocker, exhibited similar behaviors as homozygous knockouts. Taken together, our results demonstrate that retrieval of context-associated memory is dependent on the Ca_v_3.2 T-type Ca^2+^ channel.

## Introduction

There are two distinct types of voltage-gated Ca^2+^ channels (VGCCs): low voltage activated (LVA) and high voltage activated (HVA) Ca^2+^ channels [1]. LVA Ca^2+^ channels are activated after small membrane depolarizations, while HVA Ca^2+^ channels require larger membrane depolarizations [2], [3], [4], [5]. VGCCs are further classified according to the duration of activated currents into T-type for transient, L-type for long-lasting and N-type for neither T-nor L-type [4], [5]. VGCCs are composed of several different subunits: α1, α2δ, β, and γ, with the α1 subunit determining the major properties of the channel. There are at least 10 genes encoding VGCC α1 subunits [4], [6]. The Ca_v_1 and Ca_v_2 gene families encode the HVA channels, whereas the Ca_v_3 family encodes the LVA T-type channels. The pore-forming subunits of T-type Ca^2+^ channels are encoded by at least three genes in humans: CACNA1G (Cacan1g in mice; α_1G_ encoded by Ca_v_3.1), CACNA1H (Cacna1h in mice; α_1H_ encoded by Ca_v_3.2), and CACNA1I (Cacna1I in mice; α_1I_ encoded by Ca_v_3.3) [4].

T-type channels are expressed throughout the body and have been implicated in a variety of physiological processes [7], [8], [9]. In particular, the neuronal α_1H_ T-type channel, which can generate low-threshold spikes that lead to burst firing and oscillatory behavior [10], [11], [12] is highly expressed in the hippocampus and the thalamus. Pathological changes in these oscillations have been implicated in a wide range of neurological disorders [13], [14], [15], [16], [17]. *In situ* hybridization analyses have also revealed that Ca_v_3.2 transcripts are highly expressed in the hippocampal region and the thalamic reticular neurons [18]. These previous findings suggest a critical role of Ca_v_3.2 in the function of the central nervous system (CNS). Ca_v_3.2 T-channels also play an important role in the peripheral nociception and neuropathic pain [19], [20], [21]. It has been shown that Ca_v_3.2 ^−/−^ mice display attenuated pain response to all acute behavioral models of pain [21]. However, the role of Ca_v_3.2 in neuropathic pain remains controversial. Bourinet et al. show that knockdown of Ca_v_3.2 reduce acute nociceptive responses and neuropathic pain induced by CCI in rat [20]. In contrast, there is no significant difference between the WT and Ca_v_3.2 ^−/−^ mice in response to SNL-induced neuropathic pain [21]. Recently, Ca_v_3.2 has been shown to play a role in the acid-induced chronic muscle pain and colonic hypersensitivity in a rat model of irritable bowel syndrome [22], [23].

The hippocampal formation is widely recognized for its central significance in multiple aspects of CNS functions such as emotional behaviors, contextual and spatial memories [24]. The cellular mechanisms underlying these activities are represented by particular synaptic properties of this structure. More specifically, long-term potentiation (LTP) is hypothesized to be the substrate for enduring information storage [25]. Studies have indicated that molecules related to Ca^2+^ signaling are involved in the hippocampal synaptic plasticity and in learning and memory [26], [27], [28], [29]. Both VGCCs and NMDA receptors are known to be important sources of Ca^2+^ influx for the induction of LTP in certain conditions [30], [31], [32]. Various L-type calcium channels have been demonstrated to be important in different forms of memories [33], [34], [35], [36], [37], [38]. However, the involvement of particular T- channel subtypes remains controversial.

Because the Ca_v_3.2 (α_1H_) T-type calcium channel is highly expressed in the hippocampus and because its role in hippocampus-dependent learning and memory remains unclear, we used Ca_V_3.2 knock-out mice and their WT littermates as controls to investigate the function of α_1H_ T-type calcium channels in hippocampus-dependent learning behaviors. Our results indicated that Ca_V_3.2 is important for various forms of context-cued memory, but not spatial-cued or trace memory.

## Results

### Ca_v_3.2 ^−/−^ mice show normal conditioned stimulus and unconditioned stimulus perceptions and locomotor function

To investigate whether losing two copies of the Cacna1h gene leads to developmental deficits that can affect performance in behavioral tasks, we first assessed the sensory and locomotor functions of Ca_v_3.2 ^−/−^ mice to exclude any impairment that may affect performance in the trace fear conditioning (TFC) and passive avoidance tasks.

**Figure 1:**
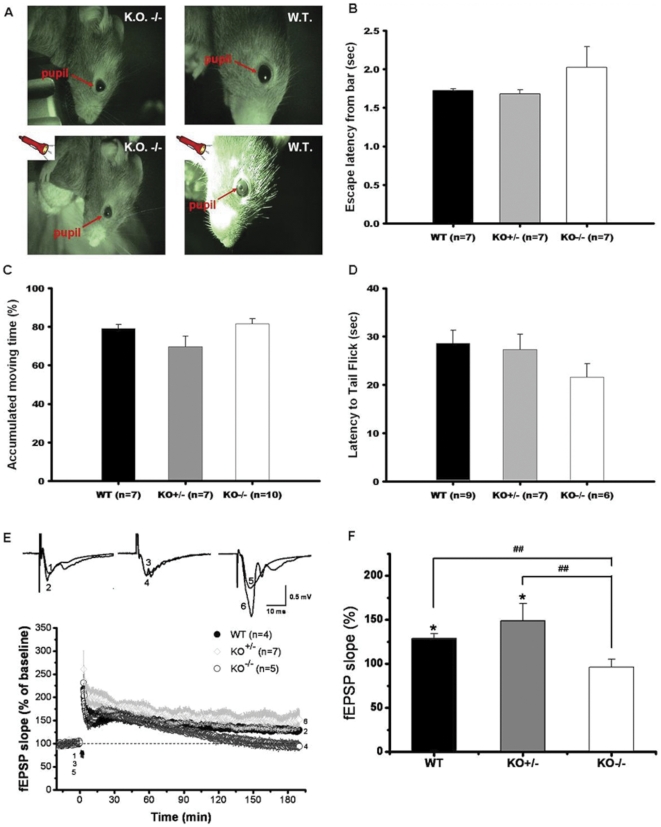
Characterization of the Ca_v_3.2 ^−/−^ mice. ***A***. Reaction to light. The reaction of the mice to light was examined by shining a bright light in the eyes and looking for the pupillary reflex. The mice were placed in a dark room for 15 minutes prior to test. The pupillary reflex of the WT and Cav3.2 ^−/−^ mice were similar. B. Visual perception test. The escape latency of the WT, Ca_v_3.2 ^+/−^ and Ca_v_3.2 ^−/−^ mice were similar when the bar was inserted to a dark chamber. Three strains stayed away from the bar and looked at it when it moved around in the air. ***C***. Locomotor activity. During the first 2 minutes of baseline and before the onset of the first tone-shock trace fear conditioning, accumulated moving times were similar among the WT, heterozygous knock-out and homozygous knock-out mice. Numbers in parentheses indicate the number of animals used in the experiment. ***D***. Nociception test. No significant difference was observed between the three groups of mice. Numbers in parentheses indicate the number of animals used in this test. (WT: wild-type; KO +/−: Ca_v_3.2 ^+/−^; KO −/−: Ca_v_3.2 ^−/−^) ***E***. LTP induced at CA1 synapse in slices from WT, Ca_v_3.2 ^−/−^ and Ca_v_3.2 ^+/−^ mice. Time course of LTP induced with high frequency stimulation. Note no significant differences in post-tetanus potentiation or expression of early LTP were found among the three groups. ***F*** *.* Bar chart showing significant differences in expression of L-LTP between WT, Ca_v_3.2 ^−/−^ and Ca_v_3.2 ^+/−^ mice. LTP values in WT, Ca_v_3.2 ^−/−^ and Ca_v_3.2 ^+/−^ mice were 128.9±5.5%, 96.3±9.1%, 149±19.5% of baseline, respectively. # # indicates *p*<0.01.

To test visual perception, we examined pupillary reflex and escape latency to an inserted bar of the Ca_v_3.2 ^−/−^ mice. The pupillary reflex to light of the Ca_v_3.2 ^−/−^ mice was similar to WT (Figure 1A). The escape latency from an inserted bar of the Ca_v_3.2 ^−/−^ mice was not significant different from that of WT and Ca_v_3.2 ^+/−^ mice (Figure 1B). During the first 2 minutes of baseline before the onset of the first tone-shock trace fear conditioning, percentage of accumulated moving time (Figure 1C) were similar between WT (78.6%), Ca_v_3.2 ^+/−^ (75.5%) and Ca_v_3.2 ^−/−^ (80.2%) mice. These results indicated that Ca_v_3.2 ^−/−^ mice have normal visual sensory function and locomotor activity.

During the initial training sessions involving tone-footshock pairings, Ca_v_3.2 ^−/−^ mice jumped and vocalized in response to the electric foot shock to a similar degree as the WT mice, suggesting normal pain perception in the mutant mice. To assess nociception in the Ca_v_3.2 ^+/−^ and Ca_v_3.2 ^−/−^ mice, the heat-induced tail-flick response was measured. The two knockout strains exhibited characteristic tail-flick responses as wildtypes in this test. No significant difference was found among the three genotypes (WT: 28.3±2.1 sec, Ca_v_3.2 ^+/−^: 27.8±2.5 sec and Ca_v_3.2 ^−/−^: 24.1±3.2 sec, Figure 1D), indicating normal pain and heat perception in the Ca_v_3.2 knockout mice.

**Figure 2:**
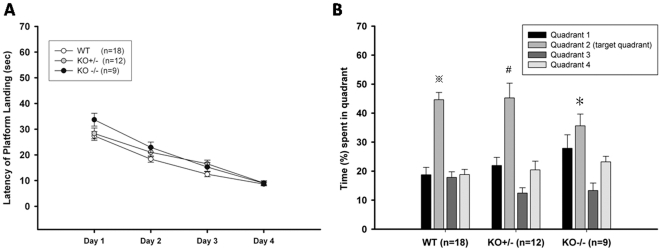
Morris water-maze tests. ***A***. Hidden platform test. No significant difference in escape time was observed between the groups each day. After four days of training, the escape time for all three groups decreased, indicating normal spatial learning and memory. ***B***. Probe trial. After training, the platform was removed from the 2^nd^ quadrant where it was originally placed in. The Y axis indicates the percentage of total time spent in one specific quadrant. The black bar, the grey bar and the white bar indicates the total time spent in non-target 1^st^, 3^rd^ and 4^th^ quadrants respectively. The light grey bar indicates the time spent in the target quadrant (the 2^nd^ quadrant). (WT: wild-type; KO +/−: Ca_v_3.2 ^+/−^; KO −/−: Ca_v_3.2 ^−/−^). Numbers in parentheses indicate the number of animals used in this experiment. <$>\raster="rg1"<$>, #, and * indicate *p*<0.05 compare with time spent in other non-target quadrants.

**Figure 3:**
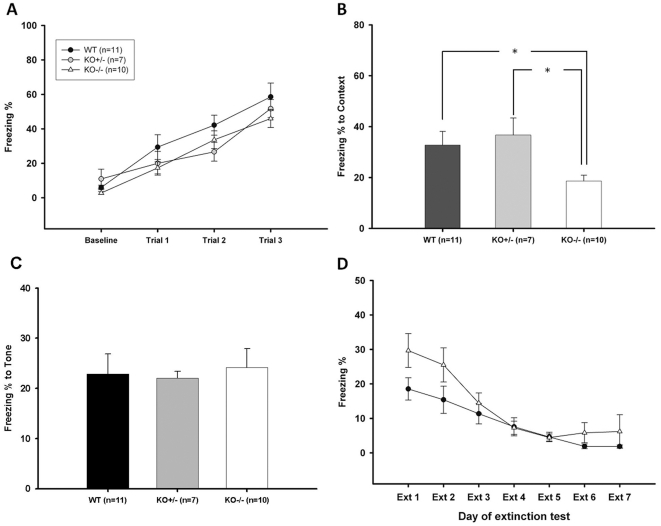
Fear conditioning tests. ***A***. The freezing percentage during the acquisition stage of the trace fear conditioning. The freezing percentage increased in all groups during the three trials of the trace fear conditioning, indicating that the mice were learning. ***B***. Contextual test. The freezing percentage for this test was significantly lower in the Ca_v_3.2 ^−/−^ mice compared to the Ca_v_3.2 ^+/−^ and WT mice 24 hours after the trace fear conditioning. Asterisk indicates *p*<0.05. ***C.*** Tone test. For this test, no significant difference in the freezing percentage was observed between the Ca_v_3.2 ^−/−^, Ca_v_3.2 ^+/−^ and WT 48 hours after the trace fear conditioning was groups. ***D***. Extinction test. After the tone test, the mice were exposed to the same tone for 7 continuous days to test for extinction. The WT (in solid circle) and homozygous knockouts (in open triangle) showed similar extinction levels after 3 days, although they responded differently on the first two days. WT: wild-type; KO +/−: Ca_v_3.2 ^+/−^; KO −/−: Ca_v_3.2 ^−/−^.

Visual function appeared normal in the Ca_v_3.2 ^−/−^ mice, as noted in the Methods. Normal performance in the hidden platform and probe trial tests of the Morris water-maze (Figure 2) further confirmed normal visual function in the knockouts. In addition, during the tone-testing phase of the trace fear conditioning, the Ca_v_3.2 ^−/−^ mice demonstrated normal freezing responses to the tone (Figure 3C), suggesting that the knock out mice perceive the tone cue normally.

### LTP at CA1 synapses in the hippocampus of Ca_v_3.2 ^−/−^ mice

To determine the extent to which the Ca_v_3.2 T-type calcium channel contributes to synaptic plasticity, we compared the expression of LTP at CA1 synapses in hippocampal slices from Ca_v_3.2 ^−/−^ and wild-type mice. No significant differences were found in the post-tetanus stimulation or early phase LTP (defined as 60 min after high frequency simulation) (Figure 1E), whereas expression of late phase LTP (defined as 180 min after high frequency stimulation) was lost in hippocampal slices obtained from Ca_v_3.2 ^−/−^ mice (Figure 1E). The magnitude of LTP of Ca_v_3.2 ^−/−^ mice was significantly lower than that of the WT mice (Figure 1F). LTP values in WT, Ca_v_3.2 ^+/−^ and Ca_v_3.2 ^−/−^ mice were 128.9±5.5%, 96.3±9.1%, 149±19.5% of baseline, respectively. We have examined the basal synaptic transmission and found no significant differences in paired-pulses ratio (Figure S1) and input/output relationship (Figure S2) between slices from Cav3.2^−/−^ and WT mice. We also tested TEA induced-LTP and found significant difference in expression pattern of this form of LTP (Figure S3).

### Ca_v_3.2 ^−/−^mice are impaired in memory retrieval for context-cued trace fear conditioning (TFC) and passive avoidance but exhibited normal memory formation and retrieval of Morris water-maze and auditory TFC

To investigate whether the Cacna1h gene is involved in spatial learning and memory, we tested the Ca_v_3.2 ^−/−^ mice in both the hidden platform and probe trial tests. The Ca_v_3.2 ^−/−^ mice learned as well as WTs in hidden platform test (Figure 2A) and spent similar percentage of time as WTs in the target quadrant in the probe trial test (WT: target quadrant (TQ) = 44.62±2.51%, Non-target quadrant (NTQ) = 18.48±1.21%; KO +/−: TQ = 45.23±5.09%, NTQ = 18.26±1.62%; KO −/−: TQ = 35.64±4.05%, NTQ = 21.45±2.16%; Figure 2B), which indicated their memory of the platform's location was as accurate as that of the other groups.

We also performed TFC tests on mice to assess their emotional and temporal-associated memory. During the initial training sessions involving TFC, both the Ca_v_3.2 ^−/−^ and WT mice exhibited comparable degrees of freezing (Figure 3A). However, when the Ca_v_3.2 ^−/−^ and WT mice were tested for conditioned trace fear to the context (in the same chamber) 24 hr after TFC, the Ca_v_3.2 ^−/−^ mice demonstrated substantially less freezing behavior in response to the conditioning chamber (*p*<0.05) (Freezing % to context: WT = 32.71±5.40%, KO +/− = 36.69±6.73%, KO −/− = 18.61±2.27%; Figure 3B).

Twenty-four hours after the contextual testing (48 hr after TFC), the same mice were tested for conditioned fear to tone in a novel chamber. When the tone was presented without the foot-shock, both the Ca_v_3.2 ^−/−^ and WT mice exhibited similar freezing responses (Freezing % to tone: WT = 22.89±3.99%, KO +/− = 22.06±1.35%, KO −/− = 24.16±3.77%; Figure 3C). These results indicate that the Ca_v_3.2 ^−/−^ mice exhibit a selective impairment in contextual trace fear learning, whereas the temporal memory associated with the tone remained intact. The memory extinction for the tone was similar between WT and Ca_v_3.2 ^−/−^ mice (Figure 3D).

**Figure 4:**
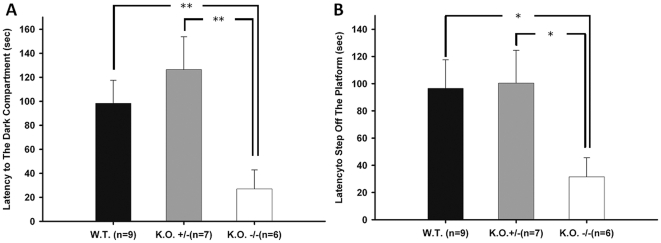
Passive avoidance tests. ***A.*** Step-through passive avoidance test. The latency of the mice entering the dark compartment was recorded 24 hours after the training session. The latency of the Ca_v_3.2 ^−/−^ mice (KO −/−) was significantly shorter than that of the WT and the Ca_v_3.2 ^+/−^ (KO +/−) mice. Asterisk indicates *p*<0.01; numbers in parentheses indicate the number of animals used in this experiment. ***B.*** Step-down passive avoidance. The latency of the mice to step off the elevated platform onto the floor was recorded 24 hours after the training session. The latency of the Ca_v_3.2 ^−/−^ (KO −/−) to step off the platform was significantly shorter than that of the WT and Ca_v_3.2 ^+/−^ (KO +/−) mice. Asterisk indicates *p*<0.05; numbers in parentheses indicate the number of animals used in this experiment.

We next performed another hippocampus-dependent passive avoidance conditioning task that included step-down and step-through tests. For the step-through test performed 24 hours after training, the Ca_v_3.2 ^−/−^ mice displayed a significantly shorter latency for entering the dark compartment compared to the WT and Ca_v_3.2 ^+/−^ mice (Latency to the dark compartment: WT = 98.33±19.07 sec, KO +/− = 126.43±27.31 sec, KO −/− = 27±15.87 sec; Figure 4A). Likewise, for the step-down passive avoidance test performed 24 hours after training, the Ca_v_3.2 ^−/−^ mice stepped off the elevated platform onto the floor more rapidly than the WT and Ca_v_3.2 ^+/−^ mice (Latency to step off the platform: WT = 96.56±20.98 sec, KO +/− = 100.43±24.11 sec, KO −/− = 31.5±14.00 sec; Figure 4B).

### Infusion of a T-type calcium channel blocker, mibefradil, into the hippocampus impaired context-cued TFC

**Figure 5:**
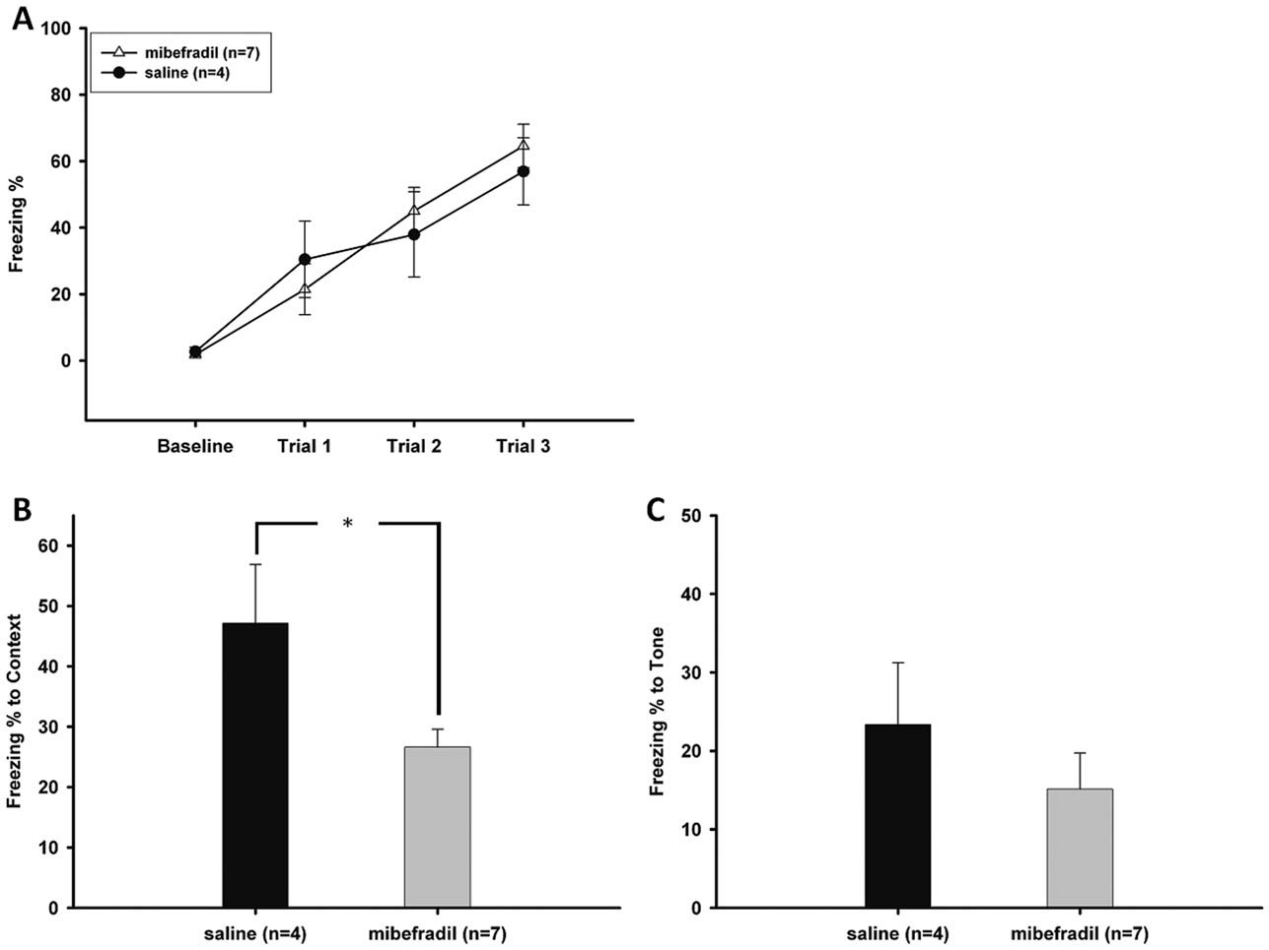
Fear conditioning tests after mibefradil infusion. ***A***. Freezing behavior after mibefradil infusion. After infusion with mibefradil, all mice appeared well groomed and healthy in their home cages and exhibited normal freezing behaviors during training courses. ***B*** *.* Context-cued trace fear memory test after infusion with mibefradil. The mice infused with mibefradil exhibited a significantly lower freezing percentage than the sham control. Asterisk indicates *p*<0.05; numbers in parentheses indicate the number of animals used in this experiment. ***C.*** Auditory trace fear memory test after infusion with mibefradil. To test the trace fear memory of the tone, the mice infused with mibefradil or saline were placed into a chamber different from the one where they had received the trace fear conditioning and the same tone cue was given for 6 minutes without subsequent electrical foot shock. No significant difference was observed between the two groups. Numbers in parentheses indicate the number of animals used in this experiment.

To further confirm the memory deficits in context-cued TFC in mice lacking Ca_v_3.2 T-type calcium channels, we infused the T-type calcium channel blocker mibefradil into the 3^rd^ ventricle near the hippocampus of WT mice to knock down the function of the local α_1H_ T-type calcium channels. This infusion was performed 3.5 hours before training so that the drug could diffuse across the bilateral hippocampi. After 24 hours, the mice received the contextual test in the same conditioning chamber. After 1 hour the contextual test had finished (25 hours after training), the mice were placed into a novel chamber for the tone test. All of the training and testing procedures were completed within 29 hours instead of the two-day protocol used in the behavioral test because the half-life of mibefradil is 40 hours. After infusion with mibefradil, all mice appeared well groomed and healthy in their home cages and exhibited normal freezing behaviors during training courses (Figure 5A).

Compared with the sham controls infused with saline, the mice infused with mibefradil exhibited significantly reduced freezing in response to the context (Freezing %: saline = 47.15±9.74%, mibefradil = 26.63±2.95%; Figure 5B) but similar freezing in response to the tone (Freezing % to tone: saline = 23.35±7.87%, mibefradil = 15.14±4.01%; Figure 5C). Because the deficit was selective for the context-cued TFC, it is unlikely to be attributable to a generalized learning defect or to health-related problems. Further, to ascertain whether the changes in the freezing response were not simply caused by altered motor performance (i.e., ability to remain motionless), other behavioral indices including locomotion, grooming, and long-body posture were also measured and no differences were found between the two groups (data not shown).

## Discussion

*In situ* hybridization studies showed that the Ca_v_3.2 gene is highly expressed in the hippocampus [18]. The hippocampus is involved in not only memory consolidation, but also the processing of spatial information, temporal information and multimodal associations. Thus it is important to determine whether this gene is involved in hippocampus-dependent learning. In the present study, we evaluated Ca_v_3.2 ^−/−^ mice in numerous behavioral paradigms. Our results showed that mice heterozygous for the Ca_v_3.2 mutation behaved similarly to WT mice in all learning and memory tasks performed in this study. This indicates that one copy of the Ca_v_3.2 gene is sufficient for these hippocampus-dependent tasks. In addition, calcium influx through other channels could compensate for the functional deficit caused by losing one copy of the Ca_v_3.2. In contrast, homozygous Ca_v_3.2 knockouts performed normally in the Morris water-maze and auditory TFC tasks, but were impaired in the context-cued TFC, step-down and step-through passive avoidance tasks. In addition, EPSP recorded from hippocampal slices showed that Ca_v_3.2 ^−/−^ mice did not express the late-phase hippocampal LTP, whereas the Ca_v_3.2 ^+/−^ mice were similar to the WT.

Calcium-dependent signaling has been shown to be important for the induction and maintenance of LTP. Calcium flow required for the induction and maintenance of hippocampal LTP can enter the cytoplasm through both NMDA receptors and postsynaptic VGCCs. Postsynaptic activation of VGCCs occurs upon theta-burst stimulation and that downstream activation of the ERK cascade is dependent upon calcium influx through L-type VGCCs [39]. The function of L-type calcium channels in learning and memory is controversial according to behavioural experiments using L-type calcium channel blockers. One investigation suggests that pharmacological blockade of L-type calcium channel enhances learning possibly through changes in cerebral blood flow [40], while other blocker-based studies indicate L-type calcium channels trigger processes that underlie the hippocampus dependent spatial memory formation [41] or amygdala-dependent conditioned fear memory [42]. Mice lacking Ca_v_1.2 in the hippocampus show a selective loss of NMDAR-independent LTP and a severe impairment of spatial memory [36], [38], [43]. On the other hand, the Ca_v_1.3 knockout mice exhibit impaired consolidation of contextual fear conditioning but normal in extinction of contextually conditioned fear and hidden platform trial of Morris water-maze [35]. In addition to L-type channels, T-type channels were also shown to be involved in LTP induction and memory formation. Blockade of postsynaptic T-type VGCCs suppressed theta-burst induced LTP [44]. T-type VGCCs blockers, mibefradil, kurtoxin and R-(-)-efonidipine, block LTP in visual cortical slices [45]. Owing to the lack of a specific subtype blocker for T-channels, it is difficult to differentiate the role of individual T channel in hippocampus-dependent learning and different phases of LTP. By recording LTP from hippocampal slices obtained from the Ca_v_3.2 heterozygous and homozygous knockout mice, we show in this study that the α_1H_ T-type calcium channel is required for the cellular mechanism of maintaining L-LTP. Our behavioral results also demonstrate that retrieval of context-associated memory is dependent on the Ca_v_3.2. In summary, different types of calcium channels appear to play distinct roles in the formation of memory. The L-type channels Ca_v_ 1.2 and Ca_v_ 1.3 are important for consolidation of spatial and contextual fear memory respectively, whereas the T-type channel Ca_v_3.2 is significant for retrieval of context-associated memory.

Because the Ca_v_3.2 ^−/−^ mice exhibited normal spatial and auditory-trace fear memory, memory formation and retrieval of these two tasks appears to have been unaffected by the impaired L-LTP in the hippocampus caused by the loss of the Ca_v_3.2 gene. One possibility is normal performance of spatial and auditory trace fear memory may simply require that hippocampal LTP be induced for a sufficient duration. Another possibility is repetitive trainings (8 trials per day for 4 days) performed in the Morris water-maze paradigm may have helped overcome the deficit of L- LTP expression *in vivo* so that the performance of spatial memory remained normal. In contrast, the abnormal L-LTP displayed by Ca_v_3.2 ^−/−^ mice was correlated with the impairment in retrieving step-down and step-through passive avoidance and contextual-cued TFC. Our results indicate that context-associated learning is dependent on the hippocampal synaptic plasticity activated by the α_1H_ T-type calcium channel encoded by the Ca_v_3.2 gene. Though the behavioral tasks performed in the present study all require the hippocampal formation at the system level [46], our results indicate that the underlying calcium-dependent signaling pathway that regulates the retrieval of context-associated memory is different from spatial and auditory trace fear memory. Mizuno and Giese (2005) reported that the formation of long-term contextual and spatial memories requires different Ca^2+^/calmodulin kinases (CaMKs). CaMKKα is required for contextual memory formation and CaMKKß is required for spatial LTM formation [47]. Further research is necessary to pair different types of calcium channels with CaMKs to delineate Ca^2+^/calmodulin signaling pathways involved in different forms of memory.

One caveat for the abnormal behavioral performances of the Ca_v_3.2 ^−/−^ mice is that the deficits could be in components of the peripheral nervous system instead of impaired cognition-related physiology in the central nervous system. Our data showed that the Ca_v_3.2 ^−/−^ mice learned the contextual TFC and the step-down and the step-through passive avoidance tasks during the acquisition stages as successfully as the WT mice. Their locomotor activity during the training stages was also normal. These results indicate that the loss of the Ca_v_3.2 gene did not affect the peripheral functions required for the animals to learn the tasks executed in this study. The experiment involving mibefradil infusion further confirmed that local blockade of T-type calcium channels in the hippocampal region can impair memory retrieval of context-cued trace fear memory. Mibefradil has been demonstrated to be able to block LTP in the visual cortex [45]. Whether the effect we observed here is due to LTP disruption by mibefradil in the hippocampus requires further experiment to verify. The total amount of mibefradil infused into the 3^rd^ ventricle near the hippocampus was 1 µl. The possibility for such small amount of solution to reach the amygdala from the infusion site is very low. Therefore, the blocking effect of the mibefradil we observed in this study should be mainly on the hippocampus. Freezing percentage to tone was lower than that to context (Fig. 5C) after infusion of mibefradil. Since the tone testing was performed one hour after contextual testing in a novel chamber, residual effects, if any, from contextual testing might affect the performance to tone but applied to both sham and drug-infused groups. It needs to note that mibefradil blocks other T-type calcium channels in addition to the channel encoded by the Ca_v_3.2. However, by coupling the results obtained from the infusion experiment with the behavioral data from the Ca_v_3.2 ^−/−^ mice, we were able to conclude that the α_1H_ T-type calcium channel plays a significant role in context-associated memory.

Taken together, our results suggest that the Ca_v_3.2 gene is critical for the expression of L-LTP and important for memory retrieval in context-cued TFC and passive avoidance tasks. In contrast, the Ca_v_3.2 gene is not required for spatial memory or memory formation and extinction in auditory TFC. On the system level, though spatial memory, TFC and passive avoidance all depend on the hippocampus, our data imply that at the least the retrieval of context-associated memories is initiated by calcium influx through α_1H_ T-type calcium channels. Mutations of human ortholog of the Ca_v_3.2 gene, CACNA1H, are associated with childhood absence epilepsy (CAE) [48], [49] and autism spectrum disorder (ASD) [50]. Our study suggests a possible role of the Ca_v_3.2 gene in the pathology of these diseases that affect cognitive functions. In addition, our results demonstrate that functional suppression of α_1H_ T-type calcium channel reduces the retrieval of context-associated fear memory. Since fear symptoms of patients with post-traumatic stress disorder (PTSD) can be elicited with exposure to the traumatic context, development of specific α_1H_ T-type calcium channel blockers might offer a novel therapeutic direction for treating PTSD.

## Materials and Methods

### Ethics statement

All protocols used in this study have been reviewed and approved by Institutional Animal Care and Use Committee of Tzu Chi University (Approval ID: 95130) and compliant with Taiwan NSC guidelines for ethical treatment of animals.

### Animals

C57BL/6J WT, male mice, originally provided from the national laboratory animal center, were purchased and maintained undisturbed in the laboratory animal center of Tzu Chi University until the behavioral tasks were performed. The Ca_v_3.2 ^−/−^ mice were generated and genotyped as described previously [51]. The heterozygous (Ca_v_3.2 ^+/−^) mice have been bred to C57BL/6J background for 7 generations. For all experiments, homozygous, heterozygous and WT littermates generated from crossing of heterozygous mice were used. All mice were trained and tested between 6 to 8 weeks old. Amplification of exon 6 was performed using PCR to genotype the individual mice before behavioral training. Animals were housed individually in plastic and metal cages with adlibitum access to food and water under a constant 12 hr light/dark cycle. All experiments were performed in a blinded manner.

### LTP recording

The WT and Ca_v_3.2 ^−/−^ mice were anesthetized with halothane and decapitated. The brains were quickly removed and placed in ice-cold artificial cerebrospinal fluid (ACSF) containing the following: 119 mM NaCl, 2.5 mM KCl, 26.2 mM NaHCO_3_, 1 mM NaH_2_PO_4_, 1.3 mM MgSO_4_, 2.5 mM CaCl_2_, and 11 mM glucose (pH was adjusted to 7.4 by gassing with 5% CO_2_ – 95% O_2_). Transverse hippocampal slices (450 µm thick) were cut with a vibrating microtome (Campden Instruments, Loughborough, UK) and transferred to an interface-type holding chamber at room temperature (27°C). The slices were allowed to recover for a minimum of 90 min and then were transferred to an immersion type recording chamber, and then perfused at 2∼3 ml/min with ACSF containing 0.1 mM picrotoxin at room temperature. To prevent epileptiform discharge of pyramidal neurons, a cut was made at the border between the CA1 and CA3 areas. A two bipolar stainless steel electrode (Frederick Haer Company, Bowdoinham, ME) and a glass pipette filled with 3 M NaCl were positioned in the stratum radiatum of the CAl area for evoking and recording of the field EPSP (fEPSP) activity, respectively. The stimulating intensity was adjusted so that 50% of the maximal response was elicited. Stable baseline fEPSP activity was evoked every 15 sec for at least 20 min, LTP was then induced using 3 trains of 100 pulses at 100 Hz with inter train interval of 60 sec, and fEPSP activity was elicited by again every 15 sec for an additional 190 min. All signals were filtered at 2 kHz using the low-pass Bessel filter provided with the amplifier (Axopatch-1D) and digitized at 5 kHz using a CED micro 1401 interface running Signal software (Cambridge Electronic Design, Cambridge, UK). The initial slopes of the fEPSP was measured for data analysis. Synaptic responses were normalized to the average of the values measured over the baseline period. The average size of the slope of the fEPSPs recorded between 170 and 190 min after the end of high frequency stimulation was used for statistical comparisons with the unpaired *t* test, and the threshold for significance was *p*<0.05.

### Vision test

The reaction of the Ca_v_3.2 ^−/−^ mice to light was examined by shining a bright light in the eyes and looking for the pupillary reflex [52]. The mice were placed in a dark room for 15 minutes prior to test, the pupillary reflex of the Ca_v_3.2 ^−/−^ mice were not different from those of the WT animals (Figure 1A). During this observation, the lens and cornea were also examined, and no obvious defects were detected. To test visual perception, a black wooden bar was inserted quickly into the home cage, and the escape behavior of the mice was observed. The escape times of the Ca_v_3.2 ^−/−^ and WT mice were similar (Figure 1B) when the bar was inserted. Both strains stayed away from the bar and looked at it when it moved around in the air.

### Trace fear conditioning (TFC) test

The mice were first divided by genotype into three groups: WT, Ca_v_3.2 ^+/−^ and Ca_v_3.2 ^−/−^. The mice were then placed into the conditioning chamber for 15 minutes per day for three days prior to training for them to adapt to the novel environment. On the training day (the 4^th^ day), the mice received three TFC trials that began with a 20 sec tone (CS), followed by a 10 sec trace period before the animal received a 1 sec foot shock (2 mA) (US), inter training interval is 1 min. The mice were then placed for 6 minutes into the same conditioning chamber for the contextual test 24 hours later (on the 5^th^ day). On the 6^th^ day, the mice were placed into a different chamber and the tone test was applied. Here the same conditioning tone was sounded for 6 minutes followed by 1 minute in the chamber without receiving any tone or foot-shock.

### Extinction test

Twenty four hours after tone testing, the mice were placed into a different chamber, allowed to move freely without any tone or foot shock for one minute, and then receiving the same tone cue as they had been given during the 6 minutes of training. The same extinction testing procedure was performed for 7 continuous days. The freezing behavior was recorded and then converted into percentage of freezing.

### Freezing behavior data collection and analysis

All training and testing sessions of TFC were video taped and the animal's behavior was recorded and analyzed with True Scan software FreezeScan 1.0 software (Clever Sys, Inc, Reston, VA, USA). Freezing behavior is identified as no motion at all except breathing. Freezing behavior was then converted into percentage of freezing according to the following formula:
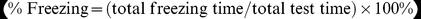



### Step-down passive avoidance test

The step-down passive avoidance task takes advantages of a mouse's desire to step-down off of a small, elevated platform (L×W×H = 10 cm×10 cm×0.5 cm) onto the metal floor of the testing apparatus (L×W×H = 30 cm×30 cm×50 cm) (manufactured by Dr. Hwei-Hsien Chen's laboratory at Tzu Chi University), and requires that the animal inhibit its behavior in order to avoid the shock. On the first day of training, the mice were shocked (2 mA, 3 sec) only after they stepped down off a small, elevated platform during training. On the next day, the mice stayed on the small platform for a longer time before stepping down; thus the normal behavior was altered. The latency to stepping down was used to assess memory.

### Step-through passive avoidance test

Mice have an innate preference for dark and enclosed environment. On the first day, the mice were handled and allowed to move freely in a chamber (30 cm×30 cm×50 cm, manufactured by Dr. Hwei-Hsien Chen's laboratory at Tzu Chi University) that was equally divided into one light and one dark compartment (30 cm×30 cm×50 cm each) for 20 minutes. On the second day, the mice were briefly confined in the light compartment first. The door leading to the adjoining darkened compartment was then opened, which allowed the animals to escape from the illuminated section of the apparatus. Once inside the dark compartment, the received a brief electrical stimulation (2 mA, 3 sec) delivered from the metal floor. On the third day, when placed inside the apparatus again, mice that recalled the shock experience in the dark compartment will avoid entering the dark during testing. Animals that exhibit longer latencies and avoidance of the dark compartment are likely to have formed hippocampus-dependent contextual memory.

### Morris water-maze

The maze is a circular pool (109 cm in diameter) filled with water mixed with non-toxic white paint. The maze was divided into four quadrants marked with different spatial cues. A round platform (10 cm in diameter) was fixed at the center of one quadrant and was hidden 1 cm beneath the water's surface. A video camera and tracking system (Track Mot, Drinstrument, Taiwan) were used to measure the escape latency. The mice were given eight trials per day for four consecutive days to find the hidden platform. The starting point for each training trial was randomly selected among the four quadrants. During each trial, each mouse was introduced to the water surface with its head pointed towards the wall of the pool. They were given 60 seconds to reach the hidden platform on their own. If they failed to do so, they were guided to the platform by gentle prodding with a stick. After they reached the platform, the mice were kept on the platform for 10 seconds. On day 5, the spatial probe trial was performed. The platform was removed from the pool and each mouse was allowed to swim freely around the maze for 60 seconds. The percentage of time spent in each quadrant was calculated. After the hidden platform and probe trial tasks, all mice were tested with a visible platform marked with a colored ball and taped above the water level that was randomly placed in each quadrant for 8 trials per day on two consecutive days.

### Stereotaxic surgery and mibefradil infusion into the hippocampus

To confirm that the memory deficit appeared in context-cued TFC of the Ca_v_3.2 ^−/−^ results from abnormal Ca^2+^ influx in the hippocampus, we infused a T-type calcium channel blocker, mibefradil, into the hippocampal formation of WT mice to knock down the function of local α_1H_ T-type calcium channels. The mice received surgery on the day before handling. Before surgery, the mice were deeply anesthetized with pentobarbital (80 mg/kg). Using a stereotaxic instrument, a cannula, each connected to a 100 µ needle was implanted in the hippocampus at the following coordinates: AP, 1.5 mm; ML, 1.8 mm (from the bregma); and DV, 1.8 mm (from the skull surface). After surgery, the mice were returned to their home cage for recovery. On the training day, the mice were divided into 2 groups: one group received an infusion of normal saline, and the other group received an infusion of mibefradil. The infusions were administered through the cannula via a 100 µl needle. In the mibefradil group, each mouse received 1 µl of 50% mibefradil (232 µl DMSO, 232 µl ACSF and 1 mg mebefradil) 3.5 hours before TFC. The mice then received the contextual test in the same conditioning chamber 24 hours later, which was followed by the tone test in a modified conditioning chamber one hour later (25 hours after TFC). Freezing behavior was recorded using a video camera and converted into percentage of freezing by FreezeScan software (Clever System Inc., Reston, VA, USA).

### Statistical analysis

All data are presented as mean ± SEM. For escape latency of light responses, fEPSP slope, accumulative moving time, hidden platform testing of Morris Water Maze, acquisition, contextual, tone, and extinction testing of fear conditioning, testing of step-through/step-down avoidance, one-way ANOVA with the variable factor for genotype was used. For probe trial test of Morris water-maze, two-way ANOVA was used with variable factors for genotype and target quadrant. An unpaired *t* test was used to analyze freezing behavior to tone and context with mibefradil treatment. Post-hoc analysis was used when there was a significant interaction between factors. Results were considered significantly different when *p*<0.05.

## Supporting Information

Figure S1 **Paired-pulse ratio.** No significant difference was recorded for paired-pulses ratio of WT (indicated in solid circle) and KO ^−/−^ (a1H^−/−^; indicated in open circle).
(TIF)

Figure S2 **Raw traces of field excitatory post synaptic potential (fEPSP).** No significant difference was observed for raw traces of fEPSP of WT (indicated in solid circle) and KO ^−/−^(a1H^−/−^; indicated in open circle).
(TIF)

Figure S3 **TEA-induced fEPSP.** No significant difference was observed for TEA-induced fEPSP of WT (indicated in solid circle) and KO ^−/−^ (a1H^−/−^; indicated in open circle).
(TIF)
