# Supplementary figures and images for: Retrieval of Context-Associated Memory is Dependent on the Cav3.2 T-Type Calcium Channel
Source: PLoS One. 2012 Jan 3;7(1):e29384. doi: 10.1371/journal.pone.0029384 (PMC3250437; doi:10.1371/journal.pone.0029384)

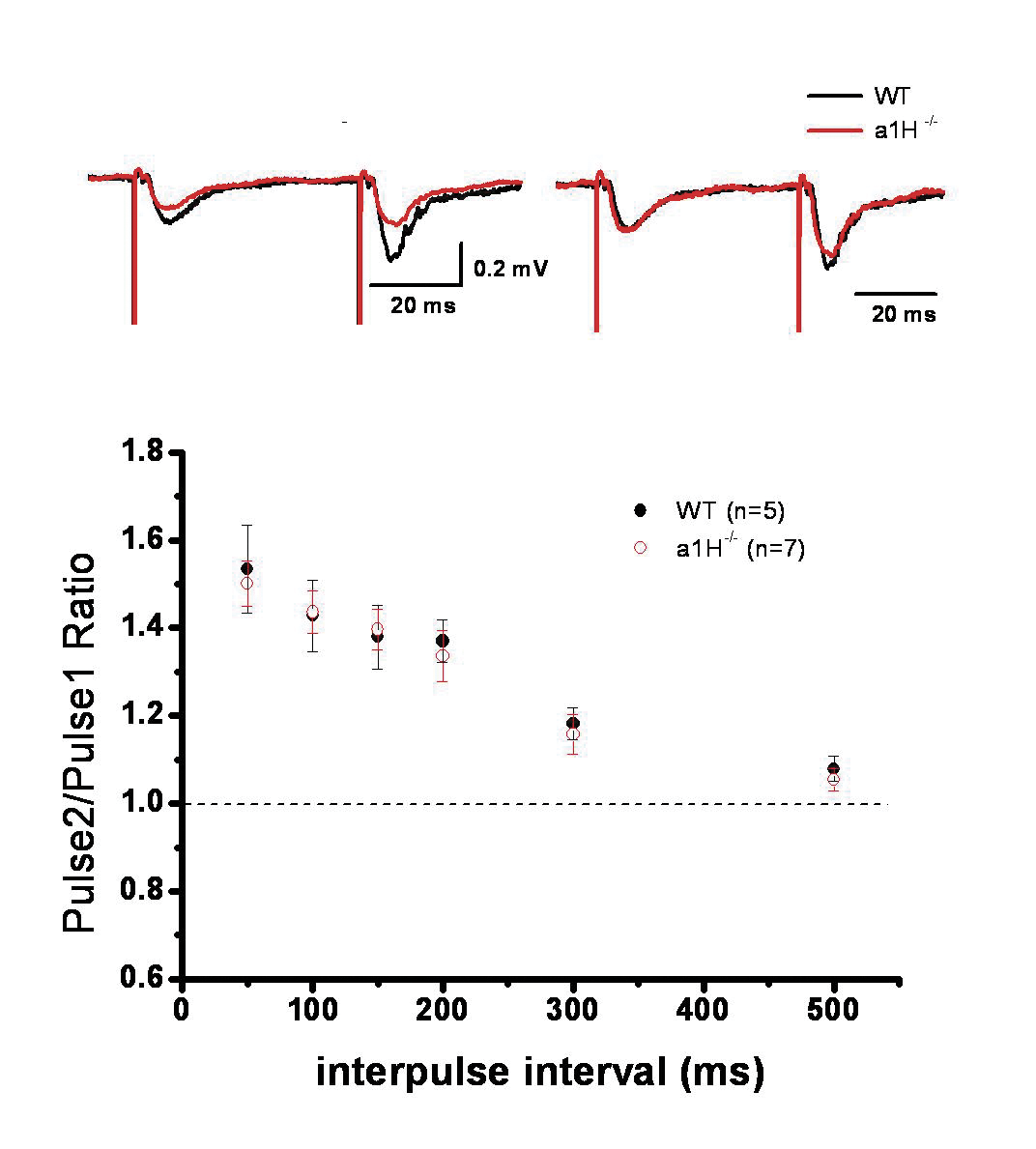

Supplement: Figure S1 — Paired-pulse ratio. No significant difference was recorded for paired-pulses ratio of WT (indicated in solid circle) and KO −/− (a1H−/−; indicated in open circle). (TIF) [file pone.0029384.s001.tif]

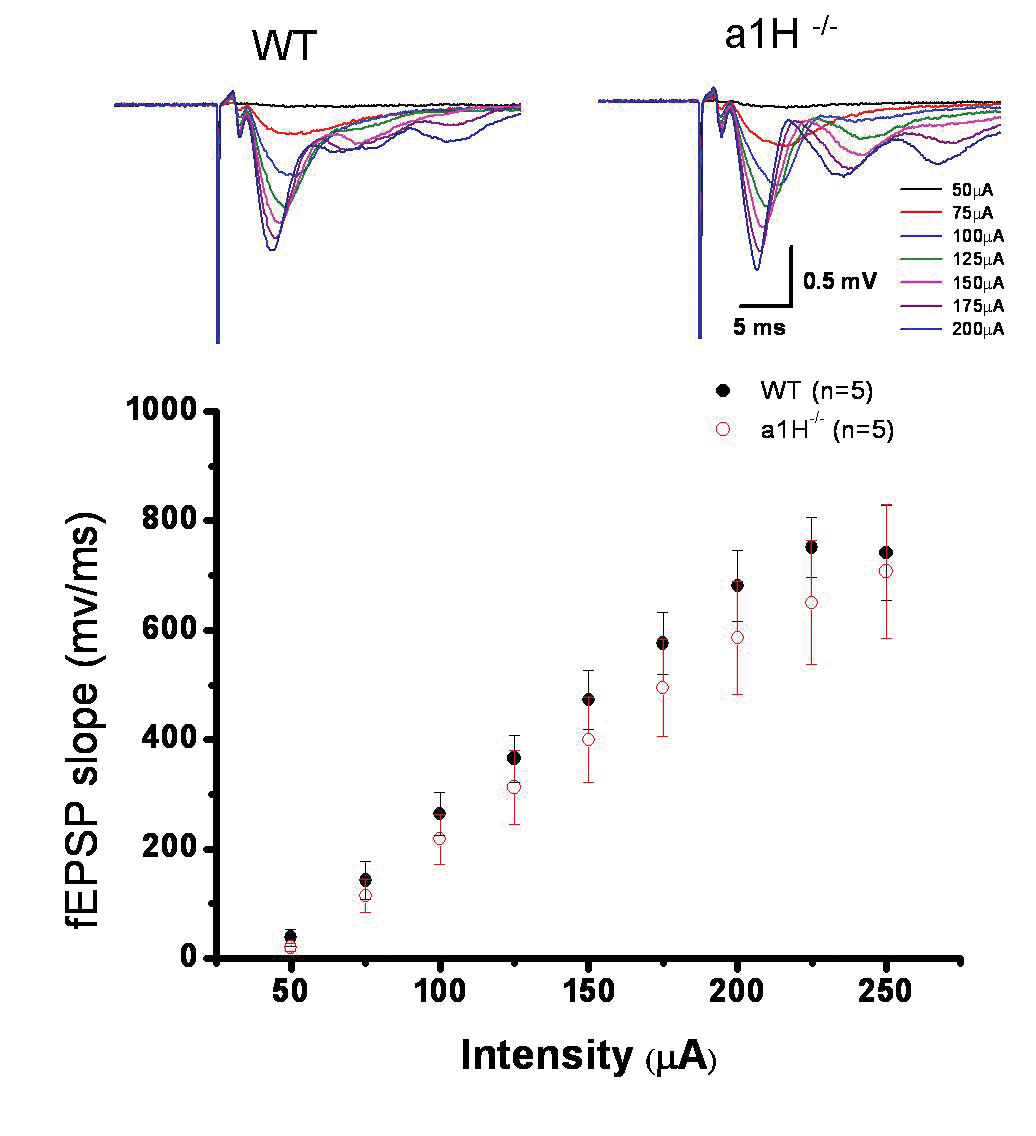

Supplement: Figure S2 — Raw traces of field excitatory post synaptic potential (fEPSP). No significant difference was observed for raw traces of fEPSP of WT (indicated in solid circle) and KO −/−(a1H−/−; indicated in open circle). (TIF) [file pone.0029384.s002.tif]

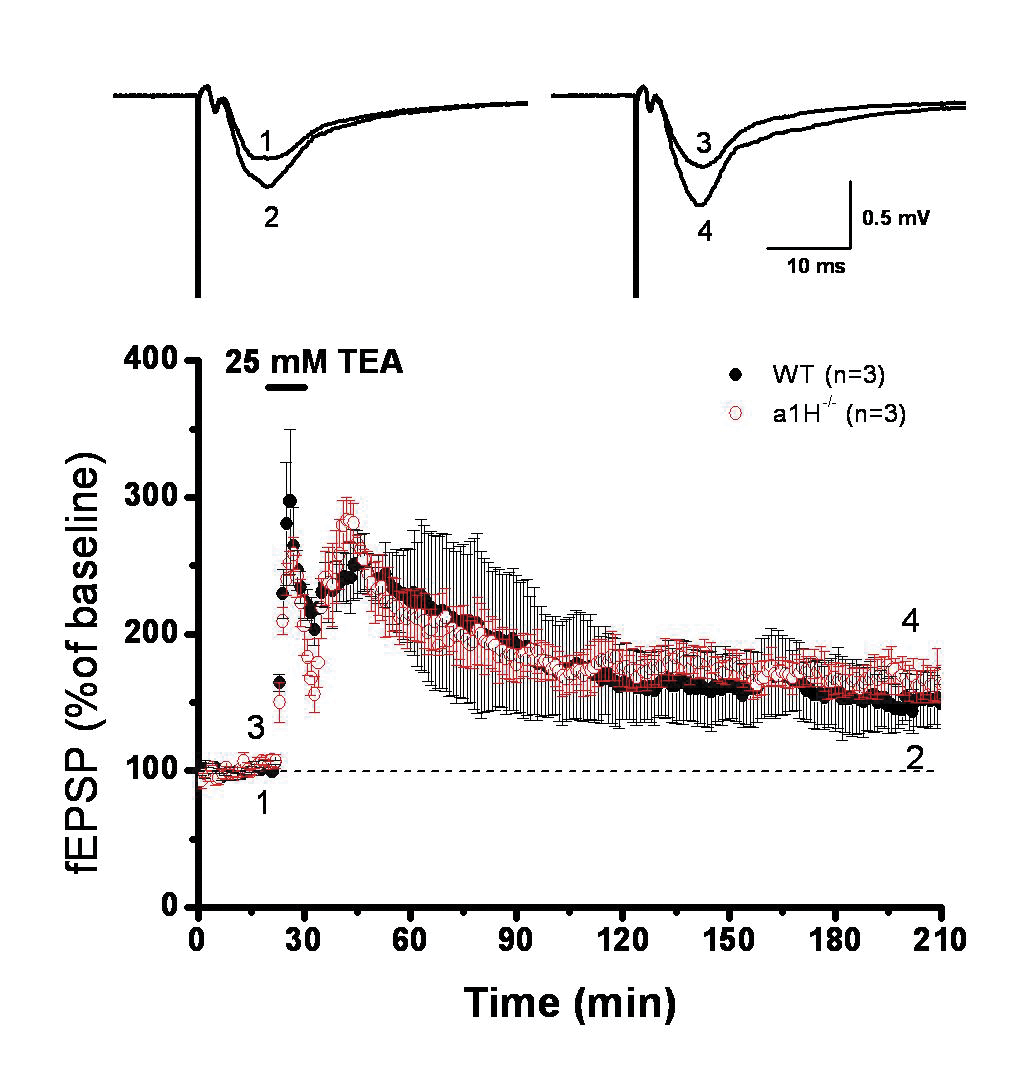

Supplement: Figure S3 — TEA-induced fEPSP. No significant difference was observed for TEA-induced fEPSP of WT (indicated in solid circle) and KO −/− (a1H−/−; indicated in open circle). (TIF) [file pone.0029384.s003.tif]
